# Competition and generalization impede cultural formation in wild jackdaws

**DOI:** 10.1098/rspb.2023.0705

**Published:** 2023-08-09

**Authors:** Josh J. Arbon, Luca G. Hahn, Guillam E. McIvor, Alex Thornton

**Affiliations:** ^1^ Centre for Ecology and Conservation, University of Exeter, Treliever Road, Penryn, Cornwall, UK; ^2^ School of Biological Sciences, University of Bristol, Tyndall Avenue, Bristol, UK

**Keywords:** culture, competition, social learning, social network, generalization, cognition

## Abstract

Animal cultures have now been demonstrated experimentally in diverse taxa from flies to great apes. However, experiments commonly use tasks with unrestricted access to equal pay-offs and innovations seeded by demonstrators who are trained to exhibit strong preferences. Such conditions may not reflect those typically found in nature. For example, the learned preferences of natural innovators may be weaker, while competition for depleting resources can favour switching between strategies and generalizing from past experience. Here we show that in experiments where wild jackdaws (*Corvus monedula*) can freely discover depleting supplies of novel foods, generalization has a powerful effect on learning, allowing individuals to exploit multiple new opportunities through both social and individual learning. Further, in contrast to studies with trained demonstrators, individuals that were first to innovate showed weak preferences. As a consequence, many individuals ate all available novel foods, displaying no strong preference and no group-level culture emerged. Individuals followed a ‘learn from adults’ strategy, but other demographic factors played a minimal role in shaping social transmission. These results demonstrate the importance of generalization in allowing animals to exploit new opportunities and highlight how natural competitive dynamics may impede the formation of culture.

## 1. Introduction

Social learning can shape individual and group behaviour through the formation of culture, with important ecological and evolutionary consequences [1–4]. The emergence of cultures has been demonstrated in numerous experiments across a range of species from flies to fish and great tits to great apes [5–8]. One commonly used approach is diffusion experiments, where novel information or behaviour is seeded into a naive population by ‘demonstrators’ that have typically been trained on one of two equal-pay-off options [8–13]. This approach provides important benefits in terms of experimental control and interpretability [9] and has been vital in demonstrating that animal culture can arise through social learning [8,10,14], but it may not necessarily reflect conditions found in the wild. The seeding of demonstrators with strong preferences, alongside the use of paradigms that reduce the effects of competition and limit the scope for generalization, may inadvertently produce conditions that are particularly conducive to the establishment of cultures. Understanding whether and how cultures form under conditions where innovations arise naturally [15] and competition can interfere with individuals' choices, as often occurs in nature, is therefore an important research priority.

Culture can emerge if innovations are socially learned and spread throughout groups. Seeding groups with trained demonstrators provides a valuable experimental tool to control the identity of innovators and the behaviour they exhibit, from which other group members can learn [8,10–13,16,17]. However, the process of training may cause these demonstrators to develop artificially strong preferences. For example, studies have trained demonstrators to avoid one of two options by making it highly distasteful [10] or unavailable during training [8]. Such reinforced preferences may therefore be stronger than those acquired through trial-and-error learning under natural conditions where stimuli can be ambiguous and sampling of alternative or unknown options may be beneficial [18]. Animals learning from these trained demonstrators are themselves more likely to adopt the strongly demonstrated preference. Indeed, in the control groups of diffusion studies where no demonstrators are seeded and innovations arise naturally, clear group-level preferences for a particular option typically fail to arise [8,17]. Moreover, if a novel behaviour is ‘discoverable’ (i.e. within the scope of the species' behavioural repertoire) it is possible that multiple innovators with different preferences may coexist [11]. Thus, compared with conditions where innovations arise naturally, trained demonstrators may provide stronger, more unambiguous models for observers to learn from, inflating the probability of arbitrary group-level culture emerging.

Another important factor that may impact the ecological realism of diffusion experiments is competition. Classical two-option diffusion experiments commonly restrict the impact of competition on learning dynamics in two key ways. First, individuals usually have an unconstrained choice between two equal options (e.g. pushing a door left or right [19]), as apparatus designs prevent individuals from monopolizing just one option. Second, many experimental designs do not feature natural resource depletion. This suppresses competition and allows unconstrained choices, whereas under natural conditions competition over access to depleting resources can favour risk-taking [20] and investigation of alternative options [21,22]. Indeed, although the discovery of alternative solutions can lead to the downfall of cultural preferences [23], experiments seldom involve scenarios where exploring alternatives is profitable (but see [22,24]). This is reasonable in the case of certain extractive foraging techniques where different, mutually exclusive methods can yield the same rewards (e.g. [25,26]). In these instances, competition is more likely to affect access to foraging items than extraction methods *per se*, and individuals will not stand to gain by performing multiple solutions. However, in many other scenarios individuals may avoid competition and maximize rewards by switching strategy. For example, vervet monkeys (*Chlorocebus pygerythrus*) followed the novel food preferences of trained demonstrators when not in competition with group-mates but switched choices when many dominants prevented access to the ‘preferred’ option [10]. Capturing natural competitive dynamics is thus vital to better understand how and when social learning leads to culture.

In addition to competition, an often overlooked factor that may influence social learning and cultural transmission is generalization: the application of learned information to new, related contexts [27]. Despite widespread evidence that animals can categorize and choose objects by their likeness to others [28], and predictions that the specificity of learning processes will impact the stability of socially transmitted behaviours [29], evidence of the effects of generalization on social learning is sparse. In naturalistic settings, generalization can be a potent tool to combat competition by allowing animals to switch to alternative options that, because of their similarity to known options, are also likely to be profitable [28]. For example, vervet monkeys that learned one method of solving a puzzle box were more likely to learn the second method [30]. However, in this example generalizing between the options did not yield a higher benefit, as both solving methods accessed the same reward. In nature, in contrast, rather than choosing *between* A and B, animals may sometimes increase their gains by choosing *both* A and B (for instance if multiple novel food types are present in the environment). Moreover, whereas Canteloup *et al.* considered the impact of prior knowledge on individual learning [30], generalization could, in principle, also affect social learning. This possibility has yet to be investigated.

**Figure 1.**
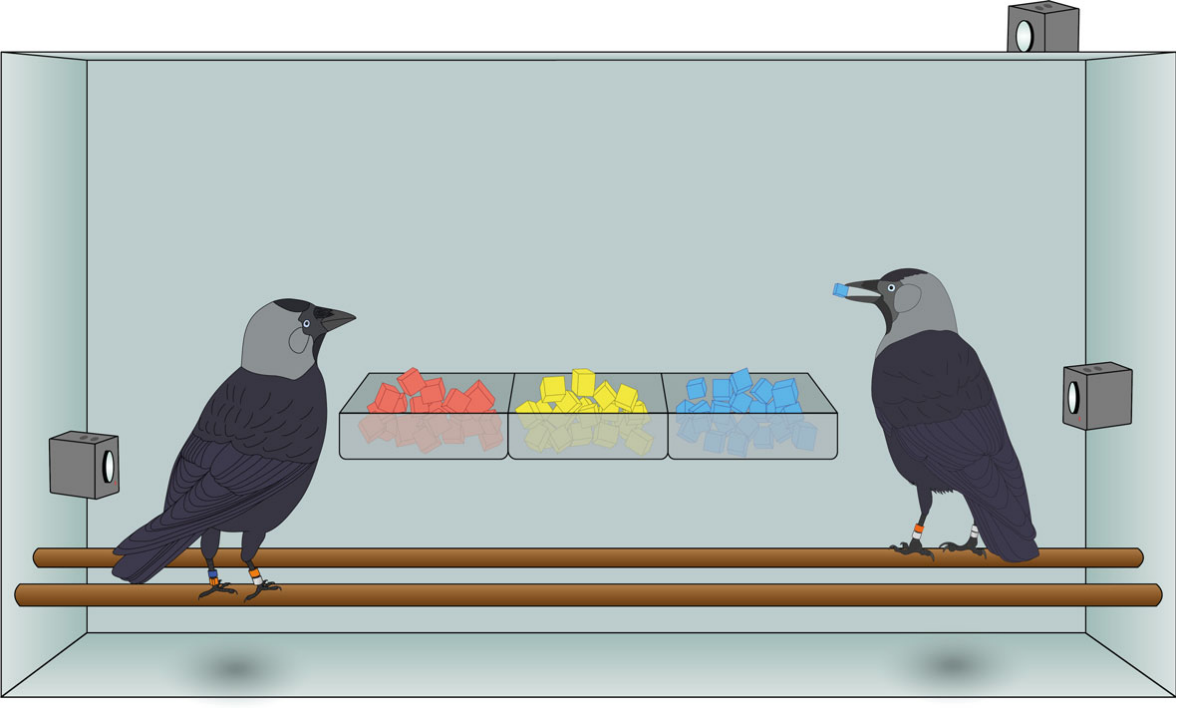
Experimental apparatus set-up. Three cameras within each arena filmed all trials of the experiment. Four different arenas were attached at a height of 3–4 m to barns and trees around the field-site.

To test how social learning and generalization interact in naturalistic settings, and how they in turn can impact cultural formation, we presented wild jackdaws (*Corvus monedula*) with two pairs of novel foods and tracked the spread of their uptake through the population. Novel foods provide a valuable high-risk, high-reward scenario to test social learning and underpin several classic experimental demonstrations of animal culture [10,31,32]. Jackdaws are known to be highly neophobic [33], but forage socially in fission–fusion flocks [34,35] and learn socially about potential risks including novel foods [36,37]. In our experiment, we used cheese dyed different colours (cf. [36]) as novel foods with generalizable characteristics (visual models confirm that the birds can discriminate between the colours used; see electronic supplementary material, figure S1). The first phase of the experiment presented blue and red simultaneously; the second phase used green and grey. These foods were identical (including in their calorific value) apart from their visual appearance, and thus represented an arbitrary choice. These novel, coloured foods, were presented alongside familiar, undyed cheese, to attract birds to the experimental arenas and enable us to assess the influence of a known food on novel food learning (figure 1). To replicate circumstances where individuals can restrict competitors' access to depleting resources, food trays were only large enough for one or two (tolerant) individuals to feed simultaneously and were not replenished when depleted. Multiple cameras filmed all feeding and observation events within the experimental arena (figure 1).

We used network-based diffusion analysis (NBDA) to estimate the effect of observing different behaviours, and the influence of generalization and individual characteristics on social learning. NBDA is a method to track the spread of learning through a population, with the key assumption that social learning follows the social network. We predicted that social learning would be a major driver in the acquisition of novel foods (following [33]), with three major pathways of social learning assessed. These pathways considered instances where birds observed others eating either (a) the same novel food of the novel pair presented (*novel-same* pathway), (b) the other novel food of the pair presented (*novel-other* pathway), and (c) known food (*familiar* pathway) (table 1). We also predicted that generalization between stimuli would greatly accelerate learning, resulting in many individuals readily eating both novel foods, with no clear cultural preference of one option over another (contrary to diffusion studies with trained demonstrators: [8,10]). A key component of these predictions is that generalization can expedite social learning as well as individual learning, such that extensive social learning does not necessarily lead to a group-level culture.

**Table 1.** The four potential learning pathways, here demonstrated for learning of blue cheese. Observation of another bird eating the stimulus on the left of ‘visualization’ leads to learning of the stimulus on the right. % represents the percentage of learning events predicted to occur through each specific pathway by the NBDA model.

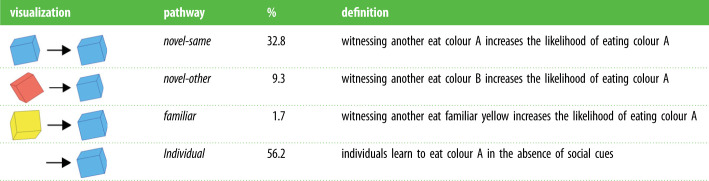

## 2. Results

### (a) Descriptive statistics

Overall, 298 individuals participated in the experiment, generating 5778 feeding bouts, which resulted in 370 unique ‘bird: novel food’ combinations encompassing all novel food colours. Of those birds, 154 were male and 105 female, with 39 unsexed. In total, 84 individuals were juveniles (fledged that year) and 214 were adults (fledged the year before or older). Competition was prevalent: individuals' choices in over 10% of feeding bouts (674/5778) were constrained, meaning that another bird blocked access to one of the options, with at least one novel colour completely depleted in 40% of trials. While solo visits to the arena were not uncommon, 60.5% of feeding events (3491/5778) had at least one potential observer. Overall, feeding events were observed by an average of 1.3 other birds, with a maximum of 13 observers for a single event.

At the population level, familiar yellow cheese was preferred to any of the novel colours. In instances where an unconstrained choice could be made, yellow was preferred to blue and red combined (binomial test: *p* < 0.001; 59.7% of feeding bouts: 1071/1794) as well as green and grey combined (binomial test: *p* < 0.001; 69.2% of feeding bouts: 1049/1515). Looking at unconstrained choices between novel colours, red was significantly preferred to blue (binomial test: *p* < 0.001; 62.3% of feeding bouts: 894/1435), with no significant preference between green and grey (binomial test: *p* = 0.544; green chosen 50.9% of feeding bouts: 668/1313).

**Figure 2.**
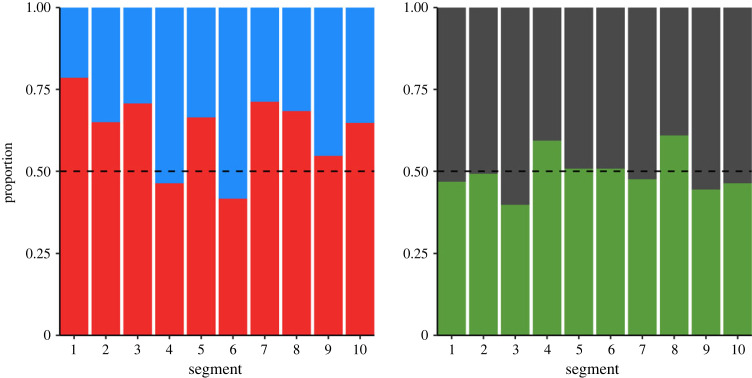
Preference plots for both novel colour dyads, with proportion of each colour eaten under free choice circumstances. Data are split into segments representing progress in the experiment; each segment is 1/10th of the feeding bouts seen. The dashed lines represent no preference.

**Figure 3.**
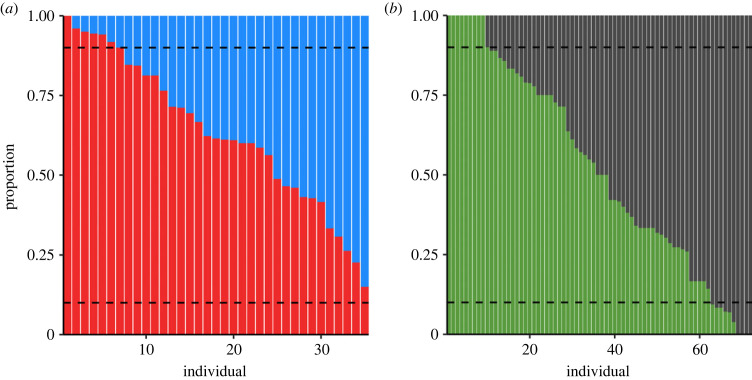
Novel food preferences of individuals that made at least 10 unconstrained choices per colour pair. Each vertical bar represents an individual that ate novel food at least 10 times with an unconstrained choice, showing the proportion of each novel colour eaten for each run of the experiment: (*a*) blue versus red; (*b*) green versus grey. Some individuals demonstrate clear preferences (e.g. individuals 1–9 in panel (*b*) only ate green), while many others ate both novel colours at relatively equal rates. Dashed lines show 0.1 and 0.9 proportions, highlighting individuals with greater than or equal to 90% preferences.

There were also no clear changes over time in preference between either of the novel colour pairs, with estimated errors larger than parameter estimates for both models (generalized estimating equation (GEE): blue versus red: estimate = −0.0213 ± 0.0233; green versus grey: estimate = −0.00171 ± 0.0223; figure 2). On an individual level, some birds ate proportionally more of one colour, but the majority did not demonstrate clear preferences. Overall, only 25/105 individuals that made 10 or more choices demonstrated a preference with a strength of 90% or larger (figure 3), a strength of preference that represents a significant deviation from random for 10 or more unconstrained choices. Indeed, the first innovators (i.e. the first bird in the population to eat blue and red, respectively) ended up preferring the opposite colour overall.

### (b) Network-based diffusion analysis

The three networks included in our NBDA (*novel-same*, *novel-other* and *familiar*; table 1) are dynamic. This means that they update with every new learning event in the network, giving a more accurate estimation for individual experience at specific time-points than aggregating the networks [38]. Each of these is analysed as a separate potential pathway through which social learning can occur, with each outputting an *S* parameter relative to its predicted strength. *S* parameters are easily interpreted by calculating the percentage of learning events that occur through each social learning pathway [38]. Individual characteristics (termed individual level values, or ILVs: [38]) can also be inputted into NBDA to estimate their effect on both social and individual (also known as asocial, or trial-and-error) learning. We included age, sex and aggression as ILVs in our model to estimate their effects on both social and individual learning. To estimate whether generalization facilitated learning, we fitted two generalization parameters as ILVs. These tested whether experience of having eaten one of the two novel foods facilitated learning of the other through (1) social or (2) individual learning. ILVs can be interpreted as having an effect on the likelihood of learning [38], such that a social learning ILV value for juveniles of 2 means they would be twice as likely to learn socially than adults, while a value of 1 represents no difference, and 0.5 half as likely. This can also be framed as learning twice/half as quickly, or individuals being better/worse social learners [38]. Our results are reported in line with the recommendations of Amrhein *et al.* [39], with point estimates for effect sizes given and discussed in relation to the potential range of effects compatible with the data (95% CIs); in all cases, point estimates are the value most compatible with the data, with those at the limits the least (for *S* parameter-likelihood profiles, see electronic supplementary material, figure S2).

### (c) Social learning pathways

**Figure 4.**
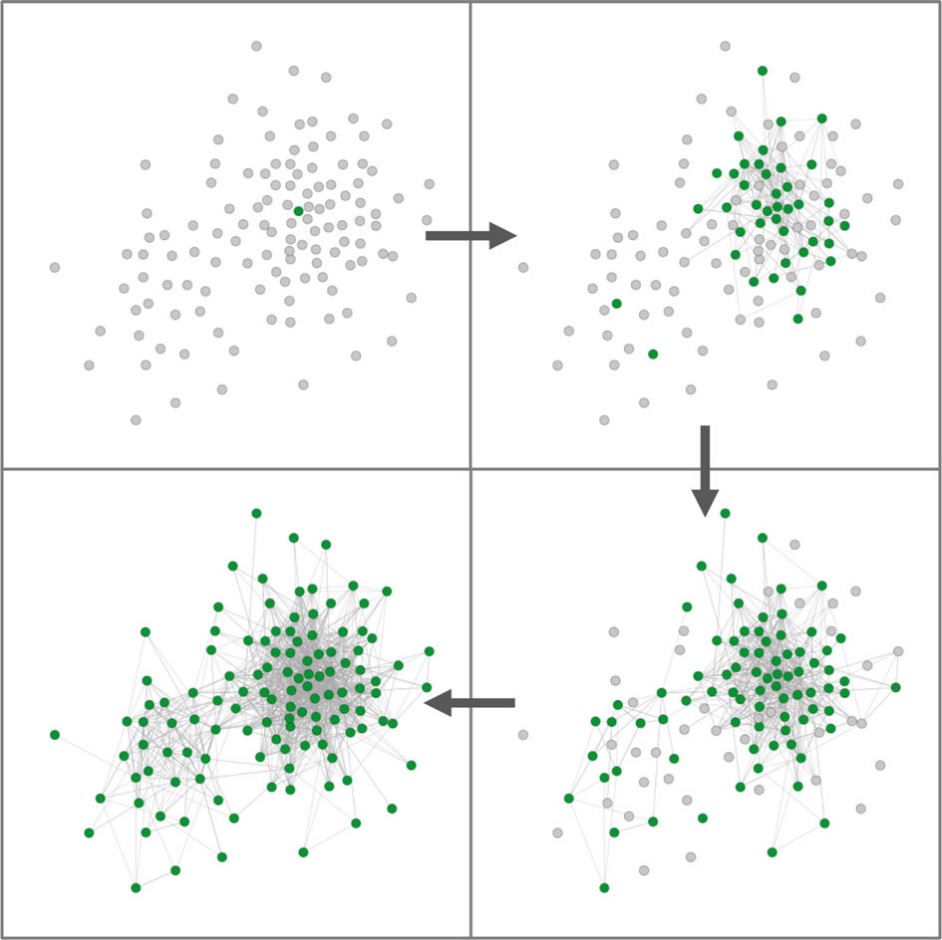
Transmission of information about green cheese through the network. Each circle represents an individual, with grey circles indicating individuals who had not eaten green cheese, turning green when they ate green cheese for the first time. Lines between individuals represent observations of individuals eating green cheese. Clockwise from top left: first individual performs behaviour, then sequentially a third more knowledgeable individuals per network. See electronic supplementary material, video S1 for a dynamic visualization.

Overall, social learning was predicted to account for 43.8% of all learning events in the experiment, with individual learning accounting for the remaining 56.2%. The largest predictor of social learning of novel food was observing others eat the same specific novel food (*novel-same: S* = 1.442, CI = 0.227–5.676; figure 4), with this pathway responsible for 32.8% of all learning events (74.9% of all social learning). There was also some evidence for generalization of social learning: individuals were more likely to learn to eat a novel food having observed a conspecific eat the other available novel colour (*novel-other: S* = 0.318, CI = 0–2.167), with an estimated 9.3% of all learning events occurring through this pathway (21.2% of social learning). We found no clear evidence that observing conspecifics eat familiar food (yellow cheese) influenced novel food learning, with only 1.7% of learning events predicted to have occurred through this pathway (*familiar: S* = 0.100, CI = 0–0.721; 3.9% of social learning). While the range of effects for both the *novel-other* and *familiar* pathway both included 0 (i.e. no social learning), comparisons of the likelihood profiles indicate that the true effect for the *novel-other* pathway is much less likely to be 0 than the *familiar* pathway (electronic supplementary material, figure S2).

### (d) Generalization from personal experience

**Figure 5.**
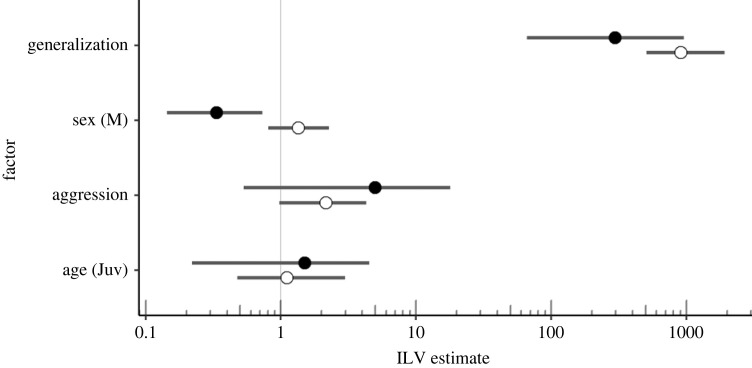
Effect size estimates for individual level values (ILVs) from the full model. Point estimates and 95% confidence intervals displayed, with black circles representing the effect of the factor on social learning, and white on individual learning. Vertical line at 1 represents no effect, with ‘sex’ representing the effect for males relative to females, and ‘age’ juveniles relative to adults.

Generalization had a strong influence on both social and individual learning. Once an individual had eaten one of the novel colours, it was far more likely to learn about the other novel colour compared with those who had not eaten the first novel colour (*Est*_individual_ = 911*x*, CI_individual_ = 144–1912*x*; *Est*_social_ = 296*x*, CI_social_ = 66–956*x*; figure 5). This indicates that individuals that had eaten one novel colour were highly likely to learn about the other, with the process predicted to affect both social and individual learning.

### (e) Individual characteristics: individual learning

The likelihood of individual learning did not differ between juveniles and adults (estimate juveniles 1.114*x* faster; CI 0.476–2.977*x*; figure 5). More aggressive individuals were estimated to be more likely to learn individually compared with less aggressive individuals, with an approximately 2*x* increase in likelihood per standard deviation increase in aggression score, although the data is also compatible with no effect or even a small negative effect of aggression (CI = 0.973–4.293*x*). Males were estimated to be 1.4*x* more likely to learn individually than females, but the data is compatible with a small negative effect of up to 1.2*x* less likely (CI = 0.809–2.275*x*).

### (f) Individual characteristics: social learning

Females were estimated to be 3*x* more likely to learn socially than males (CI = 1.364–8.772*x*). Juveniles were estimated to be 1.5*x* more likely to learn socially than adults, but this effect is inconclusive as the data is compatible with the range of this effect being from 4.5*x* less likely to 4.5*x* more likely. There was some indication that aggressive individuals were more likely to learn socially, with a 5*x* increase in likelihood per standard deviation increase in aggression score. However, the range of potential effects compatible with the data here too crosses no effect (CI = 0.530–17.850*x*; figure 5).

### (g) Age-partitioned networks

To test for an age-based social learning strategy, observation networks 1 and 2 (*novel-same* and *novel-other*) were combined, then partitioned into two new networks: (a) observations of adults and (b) observations of juveniles. Observations of adults was a far more supported pathway for social learning than observations of juveniles; learning from adults was estimated to account for 42.3% of all learning events (*S* = 8.601; CI *=* 3.616–22.146), with learning from juveniles having a negligible effect on the acquisition of new foods, predicted to be responsible for just 2% of learning (*S* = 0.103; CI = 0.38–1.86). This suggests that both adults and juveniles learned from adults in 95% of social learning events.

## 3. Discussion

Here we demonstrate that despite strong evidence for social learning effects, local cultural preferences for specific novel foods did not arise. Generalization of knowledge from personal experience and from observing others, combined with weak preferences of early adopters, probably explains this finding. Jackdaws showed a strong preference for a familiar over novel food, demonstrating their intrinsic risk aversion [40]. Nevertheless, the naturalistic design of the experiment created conditions in which generalization between stimuli was beneficial, with individuals readily eating both novel colours in each pair, subsequently providing social learning opportunities for others to do so. We therefore highlight important evidence that prior knowledge of similar stimuli can not only facilitate individual learning [30], but also social learning of a new food.

In contrast to experiments with trained demonstrators, we found that multiple individuals took the risk of innovating by sampling new foods. Indeed, although social learning was clearly evident in the experiment, individual learning played a major role. Early innovators’ preferences were also relatively weak and unstable. For instance, the first individuals to innovate eating blue and red, respectively, ended up preferring the other novel colour overall and the first innovation of blue was from an individual that had already eaten red. This is likely to have contributed to the common adoption of both novel foods by many individuals early in the experiment, stunting the formation of group-level preferences. However, we note that such conditions may not always impede the emergence of culture. For instance, in a laboratory experiment where naive bumblebees (*Bombus terrestris*) were exposed to demonstrators trained to perform two different solutions to a novel foraging task, one option still spread to became more common [14]. It is also important to recognize that the ease (or difficulty) of learning different options will influence the likelihood of adoption, which in turn may affect the potential for arbitrary cultural preferences to emerge. Taken together, this highlights the importance of understanding the strength of preferences seeded into populations by innovators, and the downstream effects of such preferences on group-level behaviour.

In our study, switching between options was also probably promoted by competition. As in naturally occurring food patches, the foods in our experiment were not replenished when depleted, and only a few individuals could access the arena at the same time. Competition therefore resulted in individuals regularly encountering situations in which not all foods were available. This led to many instances where individuals could choose to either not eat (as their ‘preferred’ option was unavailable because it was blocked or depleted) or eat a potentially ‘less-preferred’ option. This opportunity to sample alternatives will provide regular feedback to individuals that other options are also profitable, probably dampening arbitrary preferences otherwise reinforced by repeated experience. Similar processes were seen in a novel food experiment with vervet monkeys [10], but in that study the ease of access to large volumes of food that did not run out is likely to have resulted in lower levels of competition, with fewer instances where sampling alternatives was a profitable strategy. It is worth noting that in a follow-up study, these originally seeded preferences were maintained by subordinates, although the mechanisms through which preferences were maintained were not certain [41]. In experiments with unequal pay-offs, suboptimal cultures are not maintained [24]. In our study, the colour options were equal in quality, but if accessing a preferred option risks conflict or is simply not feasible, maintaining a cultural preference for this colour may itself be suboptimal. Considering the effect of competitive dynamics is therefore integral to investigating when innovations can produce stable arbitrary cultures.

The opportunities to switch presented by competition can be facilitated by generalization. We found that knowledge of one of the novel foods resulted in a large increase in probability that individuals would learn to eat the other, through a facilitation of both social and individual learning processes. This strong evidence for generalization between stimuli, with the potential that observing a conspecific eating one colour facilitated the learning of another, shows that jackdaws can apply relevant information to a new yet related scenario. Had the birds been using a simple location- or stimulus-based enhancement rule [42] where individuals were prompted to eat novel foods by watching others eat familiar foods in the arena, the *familiar* social learning pathway would have been well supported. The fact that this pathway had a negligible impact suggests instead that a generalizable characteristic of the novel food was important for social learning and that individuals that learned socially treated the novel foods as being distinct from the familiar food. For example, individuals may distinguish between familiar and novel food, but then generalize that ‘colourful food is safe to eat’ on the basis of the physical properties of the novel stimuli. Generalization could have also reduced the likelihood the jackdaws classified the other new food as novel, reducing the effects of neophobia, specifically dietary conservatism [43,44], in turn facilitating both individual and social learning. These are not mutually exclusive alternatives. Regardless of the specific pathway, this strong generalization enabled the learning of multiple options by many individuals, with learning of a first colour facilitating subsequent learning of other options. This provided regular social learning opportunities of both novel options, regardless of the original novel option adopted. This demonstrates a pathway through which consumption of both novel foods became prevalent, therefore preventing arbitrary preferences being reinforced.

The generalization abilities demonstrated in this study are likely to be beneficial in a changing world [45], where behavioural plasticity enables species resilience in the face of anthropogenic change [46,47]. Where animals will face many new but related informational challenges, the ability to draw on already acquired information will be a potent tool. In the case of jackdaws, a species prominent in anthropogenically altered habitats [48], regular foraging from anthropogenic refuse will be facilitated by the ability to generalize similar safe stimuli. In this study, we only varied the colour of the stimulus, with all other characteristics identical, which may not be entirely realistic, or at least does not fully explore the scope of potential stimuli. The degree to which generalization is employed, the breadth of stimuli considered to have shared characteristics, will have evolved in response to the specific costs and benefits of generalizing [49]. For example, generalizing about novel foods may be risky in environments where toxic items are common, and large levels of generalization are likely to be maladaptive in highly heterogenous environments [50]. As such, furthering our understanding of where generalization allows animals to switch between similar options is imperative to improving our understanding of the formation of cultures. For example, if switching between options was more risky or cognitively demanding, one might predict that arbitrary cultures would be more likely to appear as individuals are less likely to adopt alternatives. This evidence of generalization across coloured stimuli also highlights the need for greater integration of social learning research with sensory ecology to better illuminate the relationship between the sensory distinctiveness of potential solutions, their acquisition, and the stability of emergent cultures.

Another key factor to consider is individual variation. The ‘learn from older’ social learning strategy present mirrors findings in other species [51,52], with adults potentially representing a reliable source of information. However, there was relatively little evidence for clear differences in learning between categories of individuals. The effect of sex on social learning was the only conclusive individual characteristic, with females more likely to be social learners than males, raising the possibility that females may be more risk-averse. The causes of this sex difference are unclear as there is no clear evidence that male and female jackdaws differ in their dispersal patterns or risk aversion [53]—factors invoked to explain sex differences in other species [13,54,55]. As our experiment overlapped with the jackdaw breeding season, it is possible that sex differences in the costs of reproduction may impact investment in social learning (cf. [56]), but further work is needed to address this possibility. The lack of a difference in likelihood of learning between adults and juveniles is in line with meta-analytic findings on social learning experiments [57]. This suggests that within-individual variation in dietary conservatism or boldness [58], as opposed to pronounced demographic differences, drive learning and risk-taking in potentially dangerous scenarios. Indeed, the wide-ranging estimates found here mirror the large individual variation found in other studies on jackdaw behaviour [59,60]. Understanding the causes and consequences of such individual differences is therefore an important priority for social learning research [58,61].

While our study highlights factors that are not typically considered in two-option diffusion experiments using trained demonstrators, this does not in any way reduce the value of such studies in furthering our understanding of animal culture. Indeed, the use of well-trained demonstrators has produced insights into learning processes that would be impossible without such tight control [62] and generated invaluable evidence that these processes can generate culture [8,10,14,17]. Instead our aim is to stimulate research and discussion into the impacts of naturally occurring patterns of innovation, competition and generalization on the outcomes of (social) learning under natural conditions. Modifications of standard two-option experimental paradigms, including simultaneous seeding of different options [14], alongside experiments without trained demonstrators and analyses of naturally occurring behaviour [26,63] will be vital in building our understanding of these issues.

Our findings provide some important insights into the conditions under which social learning may or may not lead to cultures in animal societies. The formation of cultures has been suggested to be facilitated by scenarios in which deviating from the group strategy imposes costs [2,64]. Similarly, a situation where a single innovation solves a problem that can be applied by all (e.g. improved foraging efficiency by lobtail feeding in humpback whales, *Megaptera novaeangliae*: [63]) is probably one conducive to cultural formation, even if the performance of new behaviours by initial innovators is relatively rare. However, when competition prevents individuals from freely adopting a novel behaviour, either through depletion or restricted access, they may seek alternative strategies, which in turn will be facilitated by generalization abilities. Taken together, our results demonstrate how the ability to generalize information can interact with learning to influence information transmission and help maintain a diversity of behaviours within populations. This may also explain why despite extensive evidence for cultural transmission of novel food preferences in controlled experiments [10,31,32] there is limited evidence for arbitrary differences in preferences for naturally occurring foods in the wild [65]. With social learning and culture increasingly recognized as important conservation tools [66,67], these results highlight the need to carefully differentiate the two in some circumstances. Our findings demonstrate the importance of naturalistic resource availability and competition dynamics in social learning experiments and illustrate how these can prevent cultures from becoming the default end point of social learning in animal societies.

## 4. Methods

### (a) Subjects and study site

We ran the experiments at the Cornish Jackdaw Project, Cornwall, UK. The project maintains a wild, colour-ringed population of jackdaws with approximately 35 nest-boxes provided at each of two sites in the vicinity of Stithians, Cornwall (Site Y: 50°11′26″ N, 5°10′51″ W, and Site Z: 50°11′56″ N, 5°10′9″ W). The two sites are separated by approximately 1.5 km, with some movement of birds occurring between sites. Each bird has a unique combination of coloured leg rings, enabling individuals to be identified visually. Owing to the long-term monitoring of the study population, life-history information such as age and sex are known for many individuals and can therefore be incorporated into analyses.

Jackdaws are highly social, colony-breeding corvids [68,69] with a generalist, omnivorous diet, which often includes anthropogenic foods [48]. They display high levels of neophobia towards novel foods and objects [33,36], form large flocks and forage socially [34,35], providing ample opportunities to learn from conspecifics [36].

### (b) Diffusion experiment

We prepared novel foods by adding food-safe dye to semi-melted mild cheddar, then cutting into 0.5 cm cubes (as in [36]). To maintain colour consistency, the ratio of dye to cheese was the same throughout all preparations. Previous work confirmed the ability of jackdaws to visually discriminate red and blue novel foods [36], and we used analysis of standardized photographs parsed through an avian visual model [70–73] to confirm they can also discriminate between green and grey cheeses (electronic supplementary material, figure S1). Only six individuals from the Greggor *et al*. study were present in the 2020 study [36]; no coloured cheese or other novel foods were presented at the study site in the intervening 4-year period, so there was no potential for any other birds to have gained relevant experience.

We ran two separate two-choice open novel food diffusions from 22 June to 31 July 2020. Firstly, we presented red and blue novel foods simultaneously for 19 days. We then repeated the procedure with green and grey novel foods. In all trials, we presented the novel foods alongside a familiar food: undyed, yellow cheese, which has routinely been used as a reward in other experiments at the site. All the food types were presented in feeding trays within an experimental arena—a 90 × 50 × 40 cm opaque, open-fronted box with two perches. The arena was designed such that only birds that were within or on top of the box could easily observe other individuals' feeding choices (figure 1). We placed four experimental arenas in elevated locations (in trees and on farm buildings), with two within each of the two nest-box colonies. To avoid any biases arising based on the orientation of novel food presentation, each novel food was placed in the opposite tray in the other arena at the same site (e.g. red on the left at arena one, red on the right at arena two). However, at site Y, only one arena received regular visits so only data from three arenas were used for analysis.

At the start of each trial, 50 g of each of the two novel foods as well as the familiar food were placed in the feeding trays and recorded for an average of 2 h. We checked arenas after one hour, and if all foods had been consumed the trial was considered complete and recording ceased. Foods were never re-stocked, and as such the potential options available to birds were variable at different times during trials. We removed any remaining food at the end of trials, with the arenas left empty until the next trial, with a maximum of one trial conducted per arena per day. Trials were run concurrently within-site, and the order of set-up between the two arenas was alternated to prevent an order effect. Similarly, we alternated the starting site to prevent order or time-of-day effects becoming systematic between sites. All trials were filmed using three action cameras (SJCAM, China) placed strategically within the arena to ensure that both behaviours and colour ring combinations could be captured at all times. To achieve this, we placed two cameras on opposite sides of the arena with a third on the roof to capture the identity of observers peering from above (figure 1).

### (c) Video decoding

Video was played in QuickTime Player and transcribed into Microsoft Excel. Video was transcribed by five independent decoders who had all been trained on a test set of videos. Once video was decoded all feeding bouts, observations and aggressive interactions were checked and verified by J.J.A. To minimize the risk of observer bias, the chronological order in which videos were decoded was randomized.

Each time a bird entered the experimental arena, time and identity was noted in order to generate a ‘per-visit’ metric for aggression. Each time a bird engaged in a feeding bout, its identity and the time of the bout were recorded. Feeding bouts were defined as the continuous consumption of a unique food. For example, a bird switching from blue to red would be given one feeding bout for each food item. Further, if the bird stopped feeding to perform another behaviour (e.g. vigilance or aggression towards another individual) before resuming feeding, the resumption would constitute a new bout. In addition, each time a bird started a new feeding bout the options of potential foods available were recorded to assess preferences. An individual was recorded as having free choice if either (a) there were no other individuals eating from, or blocking access by standing in the way of or adjacent to, any other colour, or (b) an individual took food from a tray where another individual was eating, when all other options were unrestricted as in (a). If neither of these conditions were met, the options were recorded as restricted and thus excluded from choice analyses. When a certain food was exhausted, it was therefore removed from the potential options, with any free choice noted between the remaining options (e.g. in most trials, familiar yellow cheese was depleted first, and thus subsequent choices would be blue versus red, or green versus grey).

For all feeding bouts, observers were recorded. Observers were defined as individuals who had the opportunity to observe the food being eaten. This included individuals who were inside the arena, were not oriented directly away from the feeding event and did not have their line of sight clearly blocked by either the feeding trays or another bird. Individuals on the roof were considered observers if they peered over the edge of the roof into the arena. Individuals were also classed as observers if they arrived within 2 s of another bird leaving with food items conspicuously in its bill. We recorded the aggressors and victims of aggressive interactions: events where a bird performed an aggressive behaviour towards another, such as pecking, physical displacement or aggressive posturing [74]. Instances where an individual defended the apparatus to prevent other birds from entering were also considered as aggressive interactions. From these data, static aggression score was calculated for each individual as the number of aggressive behaviours per visit to the apparatus.

### (d) Analysis

All data analysis was performed in R version 4.1.1 [75], with analysis only considering birds that could be visually identified. To assess whether individuals preferred particular novel foods, we ran population-level binomial tests, considering all instances in which individuals had a free choice between (A) familiar yellow versus novel foods, and (B) between the novel food pairs (blue versus red or green versus grey). To assess if these preferences changed over time, a generalized estimating equation model was run for each food pair, using the package *gee* [76]. Data were split into 10 equal segments (by number of feeding bouts), as a proxy for progression of the experiment, with the proportion of novel food a relative to novel food b chosen used as the response. Individual ID was fitted as the clustering term, with an exchangeable correlation structure used. Subsequently, individual preferences were assessed for individuals that had eaten at least 10 novel foods within a novel food pair when free choice was available.

Data were then analysed using NBDA, using the *NBDA* package (version 0.9.6) [38], based on analyses conducted in [30]. We used the order of acquisition variant of NBDA (OADA), where the order that individuals acquire knowledge is compared with their observations from the social network, which in this case was a dynamic observation network comprising all observations of food consumption (figure 4). Each observation network updated after each learning event to reflect all observations to that time point. Owing to the semi-connected nature of the two field-sites, with some birds being seen at both, data from the two sites were compiled and analysed together, with one model for blue/red options and one for green/grey. To test for social learning of specific novel food choices (i.e. does witnessing colour A being eaten increase the probability an individual will eat colour A?), a *novel-same* observation network was used, comprising all observations of individuals witnessing the same colour being eaten. To test for the possibility of generalization of social learning (i.e. does witnessing colour A being eaten increase the probability of eating colour B?), a *novel-other* observation network was included, comprising all observations of individuals witnessing the other colour being eaten. To test whether witnessing familiar food being eaten in the proximity of novel food influenced learning, akin to a local enhancement or social facilitation effect [77], a *familiar* observation network of observations of others consuming yellow cheese was included.

For NBDA analysis all unique feeding bouts within a visit to the arena were collapsed, with observers pooled. To test for generalization of knowledge, an individual level value (ILV) was included to represent whether the individual had already eaten the other novel food, similar to the otherOpt metric in [30]. However, in contrast to previous work which had only considered the impact of generalization on individual learning, we modelled this variable to impact both social and individual learning. Age (adult versus juvenile), sex and aggression score were included as ILVs that could affect both social and individual learning. As sex data was not available for all individuals, males were coded as −0.5 and females as 0.5 with unsexed individuals set to 0. To test whether jackdaws used an age-biased learning strategy, we partitioned the observation network into observations of adults and observations of juveniles [78] and compared their relative importance. All models were fitted with individual ID as a random term, with model start values of 0 used for all parameters to aid convergence.

For the main analysis, we present a global model including all our *a priori* defined parameters of interest. One model was also run with each term of interest independently removed in order to assess its effect on model fit. In no case did the removal of any term result in an increase in model fit (as assessed using ΔAICc—electronic supplementary material, table S1). We also compared our full model with an asocial model, where each *S* parameter was constrained to be 0, in order to compare the full model including social learning with one where no social learning took place. Inference was therefore conducted from the global model [79]. We also compared our full model with an asocial model, where all social parameters are constrained to have no effect on learning (electronic supplementary material, table S1). The 95% confidence intervals for point estimates were obtained from profile log-likelihoods as suggested in Hasenjager *et al.* [38].

## Data Availability

The data are provided in the electronic supplementary material [81].
